# Social Anxiety in Victimization and Perpetration of Cyberbullying and Traditional Bullying in Adolescents with Autism Spectrum Disorder and Attention-Deficit/Hyperactivity Disorder

**DOI:** 10.3390/ijerph18115728

**Published:** 2021-05-26

**Authors:** Tai-Ling Liu, Ray C. Hsiao, Wen-Jiun Chou, Cheng-Fang Yen

**Affiliations:** 1 Department of Psychiatry, School of Medicine, College of Medicine, Kaohsiung Medical University, Kaohsiung 80708, Taiwan; tlliu@kmu.edu.tw; 2 Department of Psychiatry, Kaohsiung Medical University Hospital, Kaohsiung 80708, Taiwan; 3 Department of Psychiatry and Behavioral Sciences, University of Washington School of Medicine, Seattle, WA 98195-6560, USA; rhsiao@u.washington.edu; 4 Department of Psychiatry, Children’s Hospital and Regional Medical Center, Seattle, WA 98105, USA; 5 School of Medicine, Chang Gung University, Taoyuan 33302, Taiwan; 6 Department of Child and Adolescent Psychiatry, Chang Gung Memorial Hospital, Kaohsiung Medical Center, Kaohsiung 83301, Taiwan

**Keywords:** adolescent, attention deficit hyperactivity disorder, autism spectrum disorder, cyberbullying, traditional bullying, social anxiety, victimization

## Abstract

Victimization and perpetration of cyberbullying and traditional bullying are prevalent among adolescents with autism spectrum disorder (ASD) and attention deficit hyperactivity disorder (ADHD). The aims of this study were to examine the role of social anxiety in victimization and perpetration of cyberbullying and traditional bullying in adolescents with ASD and ADHD in Taiwan. A total of 219 adolescents with ASD and 287 adolescents with ADHD aged 11–18 years and their caregivers were recruited from the child psychiatry outpatient clinics into this study. The associations of social anxiety with victimization and perpetration of cyberbullying and traditional bullying were examined using logistic regression analysis. The results indicated that after the effects of sex, age, and autistic social impairment were controlled, social anxiety increased the risk of being a victim of cyberbullying (Odds Ratios (OR) = 1.048; 95% Confidence Interval (CI): 1.013–1.084), a victim of traditional bullying (OR = 1.066; 95% CI: 1.036–1.097), and a perpetrator of traditional bullying (OR = 1.061; 95% CI: 1.027–1.096) in adolescents with ASD. After the effects of sex, age, and ADHD symptoms were controlled for, social anxiety increased the risk of being a victim of traditional bullying in adolescents with ADHD (OR = 1.067; 95% CI: 1.039–1.096). Social anxiety was significantly associated with several forms of bullying involvement in adolescents with ASD and ADHD and warrants being considered into prevention and intervention programs for bullying involvement.

## 1. Introduction

### 1.1. Traditional Bullying and Cyberbullying Involvement in Children and Adolescents with Autism Spectrum Disorder and Attention Deficit Hyperactivity Disorder

Involvement in traditional bullying, such as physical, verbal, and relational bullying, is prevalent among children and adolescents with autism spectrum disorder (ASD) and attention deficit hyperactivity disorder (ADHD). A meta-analysis reported that the pooled prevalence estimates for victimization and perpetration of traditional bullying in students with ASD were 67% and 29%, respectively [1]. A review study also found that children with ASD are bullied by peers at a rate three to four times that of nondisabled peers with negative impacts on academic functioning and mental health symptoms, including increased risk for suicidality [2]. Especially, bullying victimization is a persistent experience for many adolescents with ASD throughout their adolescence [3]. Children and adolescents diagnosed as having ADHD or severe ADHD symptoms are also at risk of becoming victims or perpetrators of traditional bullying [4,5,6]. Involvement in traditional bullying can compromise the mental health of children and adolescents. For example, traditional bullying victimization prospectively predicts the internalization of mental health problems [7], school refusal [8], and suicidality [9] in children and adolescents with ASD. Both victims and perpetrators of traditional bullying with ADHD are more likely to experience pain, pain-related dysfunction [10], and depression [11] than their counterparts with ADHD not involved in bullying are. Traditional bullying victimization could even increase the risk of psychotic experiences among children with ADHD [12]. The results of these studies have indicated that children and adolescents with ASD and ADHD require close monitoring to minimize the possibility of involvement in traditional bullying.

Cyberbullying is a new challenge for children and adolescents in the digital age [13]. Research indicated that the time an adolescent spends using digital technologies is a powerful predictor for the risk of cyberbullying victimization [14]. Because both children and adolescents with ASD [15] and ADHD [16] spend more time on the internet compared with their counterparts without ASD and ADHD, respectively, their risk of cyberbullying victimization is increased [17,18,19]. Cyberbullying victimization markedly increases the risk of depression in adolescents with ASD [20,21,22] as well as the risk of depression and suicidality in adolescents with ADHD [18]. Therefore, cyberbullying involvement among children and adolescents with ASD and ADHD must be afforded the attention of mental health and education professionals.

### 1.2. Role of Social Anxiety in Traditional Bullying and Cyberbullying Involvement

Social anxiety is characterized as a persistent fear of social or performance situations in which an individual is exposed to unfamiliar people or possible scrutiny by others [23]. Individuals with severe social anxiety anticipate being unable to impart a positive impression on others [24] or worry about being negatively evaluated by others in social contexts [25]. Prospective studies [26,27], recall studies [28,29], and cross-sectional studies [30,31,32] have demonstrated the association between social anxiety and victimization in traditional bullying among children and adolescents in general. Perpetrators of traditional bullying are hypothesized to target socially anxious peers because they have less developed social and communication skills and are less able to defend themselves [33]. Repeated victimization in traditional bullying may increase their already high levels of social anxiety, forming a vicious circle [34]. Research has also demonstrated a positive association between adolescents’ social anxiety and cyberbullying victimization [30,35,36,37]

The association between social anxiety and the perpetration of traditional bullying and cyberbullying has received less attention in studies compared with victimization in traditional bullying and cyberbullying [27]. A prospective study determined that traditional bullying perpetration predicted higher subsequent levels of social anxiety [27], whereas a cross-sectional study revealed that perpetrators of traditional bullying had a lower level of social anxiety compared with those who were uninvolved [32]. Another study demonstrated that cyberbullying perpetrators reported social anxiety scores equal to those of individuals not involved in cyberbullying [38].

### 1.3. Role of Social Anxiety in Traditional Bullying and Cyberbullying Involvement Among Adolescents with ASD and ADHD

Social anxiety is prevalent in individuals with ASD [39] and ADHD [40]. However, the role of social anxiety in traditional bullying and cyberbullying involvement among adolescents with ASD or ADHD has not been thoroughly examined. Research indicated that victimization in traditional bullying based on the reports of adolescents’ parents correlated significantly with social anxiety symptoms in adolescents with high-functioning ASD [41]. Another study proposed that social anxiety may develop secondary to childhood ADHD following experiences of repeated criticism, insults, humiliation, and bullying [42]. However, no study has reported the association of social anxiety with the perpetration of traditional bullying or involvement in cyberbullying among adolescents. Further study is warranted to examine whether social anxiety plays a role in traditional bullying and cyberbullying victimization and perpetration independent of ASD and ADHD symptoms. If anxiety does play a role, the treatment of social anxiety in adolescents with ASD and ADHD may confer benefits in terms of the prevention and intervention in traditional bullying and cyberbullying [41].

### 1.4. Aims of the Study

The aims of this study were to examine the association of social anxiety with victimization and perpetration of traditional bullying and cyberbullying in adolescents with ASD and ADHD. Based on the results of the aforementioned studies, we hypothesized that social anxiety is significantly associated with victimization and perpetration in traditional bullying and cyberbullying in adolescents with ASD and ADHD. Because of the potentially different predictors of involvement in cyberbullying and traditional bullying [43] and the different psychopathological characteristics of ASD and ADHD, we hypothesized that the role of social anxiety differs across various types of bullying involvement and diagnoses.

## 2. Methods

### 2.1. Participants

The methods of recruiting adolescents with ASD and ADHD and their caregivers have been described in detail elsewhere [11,44]. In brief, 219 adolescents aged 11–18 years diagnosed as having ASD according to the *Diagnostic and Statistical Manual of Mental Disorders, Fifth Edition* [23] were consecutively recruited into this study from the child psychiatry outpatient clinics of three university-affiliated teaching hospitals in Taiwan between August 2013 and July 2016. In addition, 287 adolescents aged 11–18 years diagnosed as having ADHD according to the *Diagnostic and Statistical Manual of Mental Disorders, Fourth Edition, Text Revision* [45] were consecutively recruited into this study from the child psychiatry outpatient clinics of two university-affiliated teaching hospitals in Taiwan between November 2012 and November 2013. At the time of recruitment, all participants studied in inclusive classrooms and not in special education rooms. Adolescents with ASD and ADHD who had intellectual disability, schizophrenia, bipolar disorder, difficulty communicating, or any cognitive deficits based on the clinical diagnosis of child psychiatrists and chart records that prevented them from understanding the study purpose or completing the questionnaires were excluded. The adolescents’ caregivers were also invited to participate in this study. Child psychiatrists also screened whether caregivers had intellectual disability, schizophrenia, bipolar disorder, or any other cognitive deficits. Caregivers who had these cognitive deficits leading to communication difficulties were excluded. The institutional review boards of Kaohsiung Medical University (KMUHIRB-20120084 and 20120206) and Chang Gung Memorial Hospital, Kaohsiung Medical Center (101–3464c and 102–1665A3) approved the study.

### 2.2. Measures for Dependent Variables

#### 2.2.1. Experiences of Cyberbullying

Adolescents reported their experiences of cyberbullying perpetration and victimization on social media (Facebook, Twitter, and Plurk) or through pictures or video clips, emails, and blogs in the previous year using a six-item cyberbullying experiences questionnaire (CEQ) [18]. Each item is scored using a 4-point Likert scale ranging from 0 (never) to 3 (all the time). The first three items for cyberbullying perpetration addressed the posting of mean or hurtful comments; the posting of upsetting pictures, photos, or videos; and the spreading of rumors online. The final three items addressed experiences of cyberbullying victimization in terms of the actions in the first three items. Cronbach’s α of the items for cyberbullying perpetration and victimization were 0.64 and 0.70, respectively. Participants with a score of 1, 2, or 3 on any item among the first or final three items were identified as self-reported cyberbullying perpetrators or victims, respectively.

#### 2.2.2. Experiences of Traditional Bullying

The adolescents reported their experiences of traditional bullying perpetration and victimization in the previous year on the 16-item Chinese version of the School Bullying Experience Questionnaire (C-SBEQ) [46,47]. Each item is scored on a Likert 4-point scale ranging from 0 (never) to 3 (all the time). The first eight items evaluate the experiences of victimization in social, verbal, and physical bullying; the final eight items evaluate the experiences of perpetrating bullying in terms of the actions included in the first eight items. Participants who assigned 2 or 3 points to any item among the first or final eight items were identified as victims or perpetrators of traditional bullying, respectively. A previous study supported the acceptable reliability and validity of the C-SBEQ [47]. The Cronbach’s α values of the items for victimization and perpetration in traditional bullying in the present study were 0.73 and 0.70 for adolescents with ASD and 0.78 and 0.72 for adolescents with ADHD, respectively.

### 2.3. Measures for Independent Variables

#### 2.3.1. Social Anxiety

In this study, adolescents’ social anxiety symptoms were assessed using the Social Anxiety subscale of the Taiwanese version of the Multidimensional Anxiety Scale for Children (MASC-T) [48,49]. The Social Anxiety subscale contains nine items scored with a Likert 4-point scale from 0 (never true about me) to 3 (often true about me). A high total score on this subscale indicates that adolescents worry about doing something embarrassing, performing in public, being called on in class, or being laughed at or ridiculed. The psychometrics of the MASC-T were examined in a community sample of 12,536 Taiwanese children and adolescents across sex and age groups; the Social Anxiety subscale has acceptable internal consistency, 1-month test–retest reliability, and discriminant validity [49]. In the current study, the internal consistency (Cronbach’s α) of the Social Anxiety subscale was 0.79 for adolescents with ASD and 0.80 for adolescents with ADHD.

#### 2.3.2. Autistic Social Impairment

Caregivers of adolescents with ASD were invited to rate their adolescents’ autistic social impairment on the 60-item Chinese version of the Social Responsiveness Scale (SRS) [50,51]. Each item is scored on a 4-point Likert scale ranging from 1 (not true) to 4 (almost always true). A high total score indicates greater autistic social impairment. The SRS effectively distinguishes between individuals with and without ASD [50,51]. Cronbach’s α was 0.88 in the present study.

#### 2.3.3. ADHD and Oppositional Defiant Symptoms

Caregivers of adolescents with ADHD were invited to rate their adolescents’ symptoms of inattention and hyperactivity/impulsivity on the short version of the Swanson, Nolan, and Pelham Version IV Scale (SNAP-IV)-Chinese version [52,53]. Each item is scored on a 4-point Likert scale from 0 (not at all) to 3 (very much). High total scores of the subscales indicate greater inattention and hyperactivity or impulsivity symptoms. The Cronbach’s α values of the inattention and hyperactivity/impulsivity subscales were both 0.91. Moreover, caregivers of adolescents with ASD and ADHD also rate their adolescents’ oppositional defiant symptoms on the subscale of the SNAP-IV. A high total score on the subscale indicates greater oppositional defiant symptoms. The Cronbach’s α values of the oppositional defiant subscale were 0.92 in adolescents with ASD and 0.91 in adolescents with ADHD.

#### 2.3.4. Demographic Characteristics

The present study examined adolescents’ age, sex, and parental marriage status (married and live together vs. divorced or separated).

### 2.4. Procedure

All participants provided written informed consent. Adolescents with ASD and ADHD were invited to complete the Social Anxiety subscale of the MASC-T, CEQ, C-SBEQ, and a questionnaire for demographic characteristics. Caregivers of adolescents with ASD and ADHD were invited to complete the SRS and SNAP-IV, respectively. Participants could consult the research assistants if they had problems completing the questionnaires. Data analysis was performed using SPSS 20.0 (SPSS Inc., Chicago, IL, USA).

### 2.5. Statistical Analysis

The adolescents’ sex, age, involvement in cyberbullying and traditional bullying, social anxiety, autistic social impairment, ADHD symptoms, and oppositional defiant symptoms were analyzed using descriptive statistics. The differences in demographic characteristics, autistic social impairment, ADHD and oppositional defiant symptoms, and social anxiety between adolescents with various types of bullying involvements were examined using chi-squared and *t*-tests. The variables with statistical significance in chi-squared and *t*-tests were entered into logistic regression analysis to examine their associations with bullying involvement. Because of multiple comparisons, a two-tailed *p*-value of <0.0125 (0.05/4) indicated statistical significance. Odds Ratios (ORs) and 95% Confidence Intervals (CIs) were used to represent the statistical significance.

## 3. Results

Table 1 presents the participants’ demographic characteristics; social anxiety, autistic social impairment, and ADHD symptom scores; and the ratios of victimization or perpetration in cyberbullying and traditional bullying. Among the adolescents with ASD, 13.7% were cyberbullying victims, 8.2% were cyberbullying perpetrators, 26.9% were traditional bullying victims, and 18.3% were traditional bullying perpetrators. Among the adolescents with ADHD, 18.5% were cyberbullying victims, 14.3% were cyberbullying perpetrators, 20.2% were traditional bullying victims, and 13.9% were traditional bullying perpetrators.

Table 2 presents the results of chi-squared and *t*-tests comparing demographic characteristics, autistic social impairment, ADHD and oppositional defiant symptoms, and social anxiety between adolescents who were involved and who were not involved in cyberbullying. The results showed that ASD victims of cyberbullying had greater social anxiety than nonvictims, whereas no difference in social anxiety between ASD perpetrators and nonperpetrators of cyberbullying was found. No difference in social anxiety between ADHD adolescents who were involved in and were not involved in cyberbullying was found. Moreover, ASD cyberbullying perpetrators were older than nonperpetrators, whereas no difference in age between ASD cyberbullying victims and nonvictims was found. ADHD adolescents who were involved in cyberbullying were older than those who were not involved.

Table 3 presents the results of chi-squared and *t*-tests comparing demographic characteristics, autistic social impairment, ADHD and oppositional defiant symptoms, and social anxiety between adolescents who were involved and who were not involved in traditional bullying. The results showed that ASD victims and perpetrators of traditional bullying had greater social anxiety than did nonvictims and nonperpetrators. ADHD victims of traditional bullying had greater social anxiety than nonvictims, whereas no difference in social anxiety between ADHD perpetrators and nonperpetrators of traditional bullying was found. Moreover, ASD perpetrators of traditional bullying had greater autistic social impairment than did nonperpetrators.

The associations of social anxiety with cyberbullying and traditional bullying involvement were further examined using logistic regression analysis. As the results of *t* tests did not show significant difference in social anxiety between cyberbullying perpetrators and nonperpetrators in adolescents with ASD or ADHD (Table 2), the associations of social anxiety with cyberbullying perpetration were not examined using logistic regression analysis. Moreover, the result of a *t*-test showed significant differences in social anxiety and autistic social impairment between ASD perpetrators and nonperpetrators of traditional bullying (Table 3), social anxiety, and autistic social impairment were entered into multinomial logistic regression to examine their associations with traditional bullying perpetration. Table 4 showed thar social anxiety increased the risk of being a victim of cyberbullying, a victim of traditional bullying, and a perpetrator of traditional bullying in adolescents with ASD. Social anxiety also increased the risk of being a victim of traditional bullying in adolescents with ADHD.

## 4. Discussion

The present study revealed that social anxiety increased the risk of involvement in all bullying categories except cyberbullying perpetration in adolescents with ASD and increased the risk of being a victim of traditional bullying in adolescents with ADHD.

### 4.1. Social Anxiety and Victimization of Cyberbullying and Traditional Bullying

The present study revealed a significant association between social anxiety and traditional bullying victimization in both adolescents with ASD and those with ADHD. Although the cross-sectional study design limited our capacity to determine the temporal relationship between social anxiety and traditional bullying victimization, a generalized vicious cycle hypothesis was formulated based on the results of other research on children and adolescents [54]. Social anxiety can interfere with the ability of adolescents with ASD and ADHD to develop connections with peers. Without fear of retaliation, bullying perpetrators tend to target those who lack supportive and protective friends; therefore, adolescents with significant social anxiety are likely to be singled out for harassment [26,30]; subsequently, repeated victimization may increase their social anxiety. In order to stop the vicious cycle, both social anxiety and traditional bullying victimization should be the targets of intervention. Skill-based interventions address making friendships, self-control, emotional expressiveness, empathy, assertiveness, and solution of interpersonal problems may reduce the risk of traditional bullying victimization [55]. Specifically, the effectiveness of the theory of mind performance training has been approved on reducing bullying victimization in children and adolescents with high-functioning ASD [56]. Regarding social anxiety, exposure-based cognitive behavioral therapy (CBT) is a well-established intervention with positive evidence of its ability to relieve social anxiety [57]. Especially, specific programs have been developed to meet specific needs of children with comorbid ASD and anxiety [58,59]. Research has also identified interpersonal skills and emotional regulation as the two effective program components of interventions to promote mental health during adolescence [60].

The present study demonstrated a significant association between social anxiety and victimization in cyberbullying in adolescents with ASD. Socially anxious adolescents lacking the adequate social skills for peer interaction in the real world may escape from their social anxieties online. However, spending much time on the internet may increase the risk of exposure to cyberbullying victimization [61] and aggravate adolescents’ social anxiety. In addition to applying specific CBT programs to reduce social anxiety in youth with ASD [58], intervention programs for cyberbullying are important. Education on cyberbullying for the adolescent, coping skills, empathy training, communication and social skills, and digital citizenship are the most frequently used intervention components [62].

However, a significant association between social anxiety and cyberbullying victimization was not noted in adolescents with ADHD. It is possible that adolescents with ADHD may have different abilities of detecting social clues on the internet, frequencies and qualities of peer interactions, and internet activities compared with those with ASD, as well as that these differences resulted in various associations between social anxiety and cyberbullying victimization between adolescents with ASD and ADHD. Further study is warranted to examine possible etiologies that may account for the disparate results.

### 4.2. Social Anxiety and Perpetration of Cyberbullying and Traditional Bullying

The present study revealed a significant association of social anxiety with traditional bullying perpetration in adolescents with ASD but not in those with ADHD. The cross-sectional design of the present study could not determine the temporal relationship between traditional bullying perpetration and social anxiety in adolescents with ASD. Research proposed that traditional bullying perpetration provokes negative reactions from others, resulting in further social exclusion and increased social anxiety for the perpetrators [27]. It is possible that the social anxiety caused by peer rejections after perpetrating traditional bullying develops only in adolescents with ASD but not in those with ADHD. However, it is also possible that social anxiety may trigger perpetration of traditional bullying in adolescents with ASD but not in those with ADHD. Further study is warranted to examine the hypothesis.

The present study noted no significant association between social anxiety and cyberbullying perpetration in adolescents with ASD or ADHD. These results are congruent with those of previous studies [27,38]. Pabian and Vandebosch proposed that cyberbullying may be more accepted than traditional bullying is among adolescents and that cyberbullying perpetrators may be less likely to experience negative reactions from victims [27]; therefore, perpetrators of cyberbullying may encounter less social rejection and anxiety.

### 4.3. Limitations

Our study has several limitations. First, the cross-sectional research design limited our ability to draw conclusions about the causal relationships between social anxiety and involvement in cyberbullying and traditional bullying. Second, we collected the data of social anxiety and bullying involvement solely from adolescents. The problem of shared-method variance resulting from a sole information source requires careful consideration. Moreover, the self-reports are the common method to detect bullying involvement in adolescents with ASD [63] and ADHD [64]. We also excluded adolescents with intellectual disability and significant cognitive impairment to reduce the possibility of misunderstanding the questionnaires. However, further information from teachers and peers may be useful to determine the accuracy of self-report on bullying involvement in schools. Third, the participants were recruited from clinical units. Therefore, the results of this study may not be generalizable to all adolescents with ASD and ADHD. Fourth, whether the associations between social anxiety and cyberbullying and traditional bullying involvement found in adolescents with ASD and ADHD found in this study exist in adolescents without ASD and ADHD warrants further study. Fifth, research has found that compared with pure victims and perpetrators (victim-perpetrators) of bullying, those who are both victims and perpetrators of bullying have worse mental health [65,66,67,68] and social function [67]. However, there were small numbers of victim-perpetrators in this study; for example, only 4.6% of adolescents with ASD were victim-perpetrators of cyberbullying. The associations of social anxiety with involvement in cyberbullying and traditional bullying warrant further study on larger samples of adolescents with ASD and ADHD.

## 5. Conclusions

Social anxiety was significantly associated with several forms of bullying involvement in adolescents with ASD and ADHD in this study. Since social anxiety, victimization, and perpetration of bullying can compromise adolescents’ health and daily lives, health professionals must routinely monitor for them in adolescents with ASD and ADHD. It is not easy for caregivers and teachers to detect bullying victimization occurring in the cyberworld; therefore, social anxiety can be used as an indicator for early detection and stopping of bullying victimization among adolescents with ASD. CBT, behavioral intervention, and theory of mind performance training that may reduce the severities of social anxiety, and bullying involvement should be integrated into intervention programs for children and adolescents with ASD and ADHD. Further study is warranted to examine possible etiologies such as the characteristics of social interactions and the contexts of internet activities and interactions that may account for the disparate results of the associations between social anxiety and bullying involvement between adolescents with ASD and those with ADHD.

## Figures and Tables

**Table 1:** Descriptive Statistics of Demographic Characteristics, Social Anxiety, Autistic Social Impairment, ADHD and Oppositional Defiant Symptoms, and Bullying Involvement in Adolescents with ASD and ADHD.

Variable	ASD (*N* = 219)			ADHD (*N* = 287)		
	*n* (%)	Mean (SD)	Range	*n* (%)	Mean (SD)	Range
Adolescents						
Sex						
Girls	27 (12.3)			36 (12.5)		
Boys	192 (87.7)			251 (87.5)		
Age (years)		13.7 (2.1)	11–18		13.2 (2.0)	11–18
Social anxiety		49.7 (11.3)	31–78		50.5 (11.8)	31–80
Autistic social impairment		154.5 (27.2)	82–223			
Inattention					12.7 (5.4)	0–27
Hyperactivity/impulsivity					8.9 (5.8)	0–27
Opposition/defiance		10.7 (6.3)	0–24		10.1 (5.8)	0–24
Bullying involvement						
Cyberbullying victims	30 (13.7)			53 (18.5)		
Cyberbullying perpetrators	18 (8.2)			41 (14.3)		
Traditional bullying victims	59 (26.9)			58 (20.2)		
Traditional bullying perpetrators	40 (18.3)			40 (13.9)		
Caregivers						
Sex						
Female	186 (84.9)			238 (82.9)		
Male	33 (15.1)			49 (17.1)		
Age (years)		43.7 (5.4)	38–64		44.2 (6.0)	33–65
Marriage status						
Married and live together	189 (86.3)			224 (78.0)		
Divorced or separated	30 (13.7)			63 (22.0)		

ADHD: attention deficit hyperactivity disorder; ASD: autism spectrum disorder; SD: standard deviation.

**Table 2:** Comparisons of Demographic Characteristics, Autistic Social Impairment, ADHD and Oppositional Defiant Symptoms, and Social Anxiety Between Adolescents Involved and Not Involved in Cyberbullying: Chi-squared and *t*-tests.

Variable	Cyberbullying Victims				Cyberbullying Perpetrators			
	Yes	No	χ^2^ or *t*	*p*	Yes	No	χ^2^ or *t*	*p*
ASD (*n* = 219)	*n* = 30	*n* = 189			*n* = 18	*n* = 201		
Sex ^a^								
Girls	5 (16.7)	22 (11.6)	0.605	0.437	1 (5.6)	26 (12.9)	0.832	0.362
Boys	25 (83.3)	167 (88.4)			17 (94.4)	175 (87.1)		
Age (years) ^b^	14.5 (2.3)	13.6 (2.0)	−2.294	0.023	15.5 (2.3)	13.5 (2.0)	−3.926	<0.001
Marriage status								
Married and live together	22 (73.3)	167 (88.4)	4.945	0.026	15 (83.3)	174 (86.6)	0.146	0.702
Divorced or separated	8 (26.7)	22 (11.6)			3 (16.7)	27 (13.4)		
Social anxiety ^b^	55.0 (10.5)	48.9 (11.2)	−2.806	0.005	55.6 (12.1)	49.2 (11.1)	−2.324	0.021
Autistic social impairment ^b^	158.0 (21.9)	153.9 (28.0)	−0.760	0.448	163.6 (24.4)	153.6 (27.4)	−1.494	0.137
Opposition/defiance ^b^	13.0 (7.2)	10.4 (6.1)	−2.106	0.036	14.0 (5.6)	10.5 (6.3)	−2.322	0.021
ADHD (*n* = 287)	*n* = 53	*n* = 234			*n* = 41	*n* = 246		
Sex ^a^								
Girls	5 (9.4)	31 (13.2)	0.573	0.449	5 (12.2)	31 (12.6)	0.005	0.942
Boys	48 (90.6)	203 (86.8)			36 (87.8)	215 (87.4)		
Age (years) ^b^	14.1 (2.4)	13.0 (1.9)	−3.395	0.001	14.6 (2.1)	13.0 (1.9)	−4.905	<0.001
Marriage status								
Married and live together	44 (83.0)	180 (76.9)	0.937 ^a^	0.333	32 (78.0)	192 (78.0)	0.000	1.000
Divorced or separated	9 (17.0)	54 (23.1)			9 (22.0)	54 (22.0)		
Social anxiety ^b^	52.9 (11.8)	49.9 (11.7)	−1.669	0.096	50.2 (12.2)	50.5 (11.8)	0.159	0.874
Inattention ^b^	12.9 (5.8)	12.6 (5.3)	−0.363	0.717	13.2 (5.7)	12.6 (5.4)	−0.683	0.495
Hyperactivity/impulsivity ^b^	8.5 (5.7)	9.0 (5.8)	0.551	0.582	8.4 (5.4)	9.0 (5.9)	0.585	0.559
Opposition/defiance ^b^	10.3 (5.3)	10.1 (5.9)	−0.251	0.802	10.8 (5.3)	10.0 (5.9)	−0.820	0.413

^a^: *n* (%); ^b^: Mean (SD); ADHD: attention deficit hyperactivity disorder; ASD: autism spectrum disorder.

**Table 3:** Comparisons of Demographic Characteristics, Autistic Social Impairment, ADHD and Oppositional Defiant Symptoms, and Social Anxiety Between Adolescents Involved and Not Involved in Traditional Bullying: Chi-squared and *t*-tests.

Variable	Traditional Bullying Victims				Traditional Bullying Perpetrators			
	Yes	No	χ^2^ or *t*	*p*	Yes	No	χ^2^ or *t*	*p*
ASD (*n* = 219)	*n* = 59	*n* = 160			*n* = 40	*n* = 179		
Sex ^a^								
Girls	8 (13.6)	19 (11.9)	0.113	0.737	6 (15)	21 (11.7)	0.323	0.570
Boys	51 (86.4)	141 (88.1)			34 (85)	158 (88.3)		
Age (years) ^b^	14.0 (2.1)	13.6 (2.1)	−1.085	0.279	13.7 (2.0)	13.7 (2.1)	−0.151	0.880
Marriage status								
Married and live together	47 (79.7)	142 (88.8)	3.012	0.083	33 (82.5)	156 (87.2)	0.598	0.439
Divorced or separated	12 (20.3)	18 (11.2)			7 (17.5)	23 (12.8)		
Social anxiety ^b^	55.6 (10.6)	47.6 (10.8)	−4.863	<0.001	56.3 (11.4)	48.3 (10.8)	−4.198	<0.001
Autistic social impairment ^b^	160.7 (28.4)	152.1 (26.5)	−2.081	0.039	164.8 (24.1)	152.3 (27.4)	−2.555	0.011
Opposition/defiance ^b^	10.7 (6.8)	10.8 (6.1)	0.071	0.944	11.9 (7.2)	10.5 (6.0)	−1.235	0.218
ADHD (*n* = 287)	*n* = 58	*n* = 229			*n* = 40	*n* = 247		
Sex ^a^								
Girls	7 (12.1)	29 (12.7)	0.015	0.903	6 (15)	30 (12.1)	0.256	0.613
Boys	51 (87.9)	200 (87.3)			34 (85)	217 (87.9)		
Age (years) ^b^	12.8 (1.8)	13.3 (2.1)	1.945	0.053	13.4 (2.0)	13.2 (2.1)	−0.761	0.447
Marriage status								
Married and live together	46 (79.3)	178 (77.7)	0.068 ^a^	0.795	34 (85)	190 (76.9)	1.311 ^a^	0.252
Divorced or separated	12 (20.7)	51 (22.3)			6 (15)	57 (23.1)		
Social anxiety ^b^	57.5 (11.4)	48.7 (11.2)	−5.301	<0.001	52.6 (13.3)	50.2 (11.5)	−1.205	0.229
Inattention ^b^	14.0 (5.7)	12.3 (5.3)	−2.099	0.037	13.6 (5.1)	12.5 (5.4)	−1.217	0.224
Hyperactivity/impulsivity ^b^	9.6 (6.3)	8.7 (5.7)	−1.026	0.306	8.6 (4.7)	9.0 (6.0)	0.330	0.742
Opposition/defiance ^b^	10.2 (5.2)	10.1 (6.0)	−0.149	0.881	10.7 (4.7)	10.1 (6.0)	−0.598	0.551

^a^: *n* (%); ^b^: Mean (SD); ADHD: attention deficit hyperactivity disorder; ASD: autism spectrum disorder.

**Table 4:** Associations of Social Anxiety with Cyberbullying and Traditional Bullying Involvement.

Variable	Cyberbullying Victims			Traditional Bullying Victims			Traditional Bullying Perpetrators		
	B (SE)	*p*	OR (95% CI)	B (SE)	*p*	OR (95% CI)	B (SE)	*p*	OR (95% CI)
ASD									
Social anxiety	0.047 (0.017)	0.007	1.048 (1.013–1.084)	0.064 (0.015)	<0.001	1.066 (1.036–1.097)	0.059 (0.016) ^a^	<0.001	1.061 (1.027–1.096)
ADHD									
Social anxiety	-	-	-	0.065 (0.013)	<0.001	1.067 (1.039–1.096)	-	-	-

ADHD: attention deficit hyperactivity disorder; ASD: autism spectrum disorder; CI: confidence interval; OR: odds ratio; SE: standard error; ^a^ Controlling for the effect of autistic social impairment.

## Data Availability

Data of this study can be obtained from Cheng-Fang Yen.

## References

[1] Park I., Gong J., Lyons G.L., Hirota T., Takahashi M., Kim B., Lee S.Y., Kim Y.S., Lee J., Leventhal B.L. (2020). Prevalence of and factors associated with school bullying in students with autism spectrum disorder: A cross-cultural meta-analysis. Yonsei Med. J..

[2] Hoover D.W., Kaufman J. (2018). Adverse childhood experiences in children with autism spectrum disorder. Curr. Opin. Psychiatry.

[3] Tipton-Fisler L.A., Rodriguez G., Zeedyk S.M., Blacher J. (2018). Stability of bullying and internalizing problems among adolescents with ASD, ID, or typical development. Res. Dev. Disabil..

[4] Thomas H.J., Connor J.P., Lawrence D.M., Hafekost J.M., Zubrick S.R., Scott J.G. (2017). Prevalence and correlates of bullying victimisation and perpetration in a nationally representative sample of Australian youth. Aust. N. Z. J. Psychiatry.

[5] Verlinden M., Jansen P.W., Veenstra R., Jaddoe V.W., Hofman A., Verhulst F.C., Shaw P., Tiemeier H. (2015). Preschool attention-deficit/hyperactivity and oppositional defiant problems as antecedents of school bullying. J. Am. Acad. Child Adolesc. Psychiatry.

[6] Murray A.L., Zych I., Ribeaud D., Eisner M. (2021). Developmental relations between ADHD symptoms and bullying perpetration and victimization in adolescence. Aggress. Behav..

[7] Rodriguez G., Drastal K., Hartley S.L. (2021). Cross-lagged model of bullying victimization and mental health problems in children with autism in middle to older childhood. Autism.

[8] Ochi M., Kawabe K., Ochi S., Miyama T., Horiuchi F., Ueno S.I. (2020). School refusal and bullying in children with autism spectrum disorder. Child Adolesc. Psychiatry Ment. Health.

[9] Holden R., Mueller J., McGowan J., Sanyal J., Kikoler M., Simonoff E., Velupillai S., Downs J. (2020). Investigating bullying as a predictor of suicidality in a clinical sample of adolescents with autism spectrum disorder. Autism Res..

[10] Yeh Y.C., Huang M.F., Wu Y.Y., Hu H.F., Yen C.F. (2019). Pain, bullying involvement, and mental health problems among children and adolescents with ADHD in Taiwan. J. Atten. Disord..

[11] Hu H.F., Chou W.J., Yen C.F. (2016). Anxiety and depression among adolescents with attention-deficit/hyperactivity disorder: The roles of behavioral temperamental traits, comorbid autism spectrum disorder, and bullying involvement. Kaohsiung J. Med. Sci..

[12] Hennig T., Jaya E.S., Lincoln T.M. (2017). Bullying mediates between attention-deficit/hyperactivity disorder in childhood and psychotic experiences in early adolescence. Schizophr. Bull..

[13] Kowalski R.M., Limber S.P. (2013). Psychological, physical, and academic correlates of cyberbullying and traditional bullying. J. Adolesc. Health.

[14] Didden R., Scholte R.H.J., Korzilius H., De Moor J.M.H., Vermeulen A., O’Reilly M., Lang R., Lancioni G.E. (2009). Cyberbullying among students with intellectual and developmental disability in special education settings. Dev. Neurorehabil..

[15] Kuo M.H., Orsmond G., Coster W., Cohn E.S. (2014). Media use among adolescents with autism spectrum. Autism.

[16] Ko C.H., Yen J.Y., Chen C.S., Yeh Y.C., Yen C.F. (2009). Predictive values of psychiatric symptoms for Internet addiction in adolescents: A 2-year prospective study. Arch. Pediatr. Adolesc. Med..

[17] Kowalski R.M., Fedina C. (2011). Cyber bullying in ADHD and Asperger Syndrome populations. Res. Autism Spectr. Disord..

[18] Yen C.F., Chou W.J., Liu T.L., Ko C.H., Yang P., Hu H.F. (2014). Cyberbullying among male adolescents with attention-deficit/hyperactivity disorder: Prevalence, correlates, and association with poor mental health status. Res. Dev. Disabil..

[19] Dawson A.E., Wymbs B.T., Evans S.W., DuPaul G.J. (2019). Exploring how adolescents with ADHD use and interact with technology. J. Adolesc..

[20] Hu H.F., Liu T.L., Hsiao R.C., Ni H.C., Liang S.H., Lin C.F., Chan H.L., Hsieh Y.H., Wang L.J., Lee M.J. (2019). Cyberbullying victimization and perpetration in adolescents with high-functioning autism spectrum disorder: Correlations with depression, anxiety, and suicidality. J. Autism Dev. Disord..

[21] Wright M.F. (2017). Cyber victimization and depression among adolescents with autism spectrum disorder: The buffering effects of parental mediation and social support. J. Child Adolesc. Trauma.

[22] Wright M.F., Wachs S. (2019). Does peer rejection moderate the associations among cyberbullying victimization, depression, and anxiety among adolescents with autism spectrum disorder?. Children.

[23] American Psychiatric Association (2013). Diagnostic and Statistical Manual of Mental Disorders.

[24] La Greca A.M., Lopez N. (1998). Social anxiety among adolescents: Linkages with peer relations and friendships. J. Abnorm. Child Psychol..

[25] Essau C.A., Conradt J., Petermann F. (1999). Frequency and comorbidity of social phobia and social fears in adolescents. Behav. Res. Ther..

[26] Acquah E.O., Topalli P., Wilson M.L., Junttila N., Niemi P.M. (2016). Adolescent loneliness and social anxiety as predictors of bullying victimization. Int. J. Adolesc. Youth.

[27] Pabian S., Vandebosch H. (2016). An investigation of short-term longitudinal associations between social anxiety and victimization and perpetration of traditional bullying and cyberbullying. J. Youth Adolesc..

[28] Boulton M.J. (2013). Associations between adults’ recalled childhood bullying victimization, current social anxiety, coping, and self-blame: Evidence for moderation and indirect effects. Anxiety Stress Coping.

[29] McCabe R.E., Antony M.M., Summerfeldt L.J., Liss A., Swinson R.P. (2003). Preliminary examination of the relationship between anxiety disorders in adults and self-reported history of teasing or bullying experiences. Cogn. Behav. Ther..

[30] Coelho V.A., Romão A.M. (2018). The relation between social anxiety, social withdrawal and (cyber) bullying roles: A multilevel analysis. Comput. Hum. Behav..

[31] Hawker D.S.J., Boulton M.J. (2000). Twenty years’ research on peer victimization and psychosocial maladjustment: A meta-analytic review of cross-sectional studies. J. Child Psychol. Psychiatry.

[32] Juvonen J., Graham S., Schuster M. (2003). Bullying among young adolescents: The strong, the weak, and the troubled. Pediatrics.

[33] La Greca A.M., Stone W.L. (1993). Social anxiety scale for children-revised: Factor structure and concurrent validity. J. Clin. Child Psychol..

[34] Craig W.M. (1998). The relationship among bullying, victimization, depression, anxiety, and aggression in elementary school children. Pers. Individ. Dif..

[35] Dempsey A.G., Sulkowski M.L., Nichols R., Storch E.A. (2009). Differences between peer victimization in cyber and physical settings and associated psychosocial adjustment in early adolescence. Psychol. Sch..

[36] Juvonen J., Gross E.F. (2008). Extending the school grounds? Bullying experiences in cyberspace. J. Sch. Health.

[37] Navarro R., Yubero S., Larranaga E., Martinez V. (2011). Children’s cyberbullying victimization: Associations with social anxiety and social competence in a Spanish sample. Child Indic. Res..

[38] Kowalski R.M., Limber S.P., Agatston P.W. (2008). Cyber Bullying: Bullying in the Digital Age.

[39] Kent R., Simonoff E., Kerns C.M., Renno P., Storch E.A., Kendall P.C., Wood J.J. (2017). Prevalence of anxiety in autism spectrum disorders. Anxiety in Children and Adolescents with Autism Spectrum Disorder.

[40] Liu T.L., Yang P., Ko C.H., Yen J.Y., Yen C.F. (2014). Association between ADHD symptoms and anxiety symptoms in Taiwanese adolescents. J. Atten. Disord..

[41] van Schalkwyk G., Smith I.C., Silverman W.K., Volkmar F.R. (2018). Bullying and anxiety in high-functioning adolescents with ASD. J. Autism Dev. Disord..

[42] Koyuncu A., Alkin T., Tukel R. (2018). Development of social anxiety disorder secondary to attention deficit/hyperactivity disorder (the developmental hypothesis). Early Interv. Psychiatry.

[43] Yang S.J., Stewart R., Kim J.M., Kim S.W., Shin I.S., Dewey M.E., Maskey S., Yoon J.S. (2013). Differences in predictors of traditional and cyber-bullying: A 2-year longitudinal study in Korean school children. Eur. Child Adolesc. Psychiatry.

[44] Chou W.J., Wang P.W., Hsiao R.C., Hu H.F., Yen C.F. (2020). Role of school bullying involvement in depression, anxiety, suicidality, and low self-esteem among adolescents with high-functioning autism spectrum disorder. Front. Psychiatry.

[45] American Psychiatric Association (2000). Diagnostic and Statistical Manual of Mental Disorders.

[46] Kim Y.S., Koh Y.J., Noh J. (2001). Development of Korean-Peer Nomination Inventory (K-PNI): An inventory to evaluate school bullying. J. Korean Neuropsychiatr. Assoc..

[47] Yen C.F., Kim Y.S., Tang T.C., Wu Y.Y., Cheng C.P. (2012). Factor structure, reliability, and validity of the Chinese version of the School Bullying Experience Questionnaire. Kaohsiung J. Med. Sci..

[48] March J.S. (1997). Multidimensional Anxiety Scale for Children.

[49] Yen C.F., Yang P., Wu Y.Y., Hsu F.C., Cheng C.P. (2010). Factor structure, reliability and validity of the Taiwanese version of the Multidimensional Anxiety Scale for children. Child Psychiatry Hum. Dev..

[50] Constantino J.N., Davis S.A., Todd R.D., Schindler M.K., Gross M.M., Brophy S.L., Metzger L.M., Shoushtari C.S., Splinter R., Reich W. (2003). Validation of a brief quantitative measure of autistic traits: Comparison of the social responsiveness scale with the autism diagnostic interview-revised. J. Autism Dev. Disord..

[51] Gau S.S.F., Liu L.T., Wu Y.Y., Chiu Y.N., Tsai W.C. (2013). Psychometric properties of the Chinese version of the Social Responsiveness Scale. Res. Autism Spectr. Disord..

[52] Gau S.S., Shang C.Y., Liu S.K. (2008). Psychometric properties of the Chinese version of the Swanson, Nolan, and Pelham, version IV scale–Parent form. Int. J. Methods Psychiatr. Res..

[53] Swanson J.M., Kraemer H.C., Hinshaw S.P., Arnold L.E., Conners C.K., Abikoff H.B., Clevenger W., Davies M., Elliott G., Greenhill L.L. (2001). Clinical relevance of the primary findings of the MTA: Success rates based on severity of ADHD and ODD symptoms at the end of treatment. J. Am. Acad. Child Adolesc. Psychiatry.

[54] Siegel R.S., La Greca A.M., Harrison H.M. (2009). Peer victimization and social anxiety in adolescents: Prospective and reciprocal relationships. J. Youth Adoles..

[55] da Silva J.L., de Oliveira W.A., Braga I.F., Farias M.S., da Silva Lizzi E.A., Gonçalves M.F., Pereira B.O., Silva M.A. (2016). The effects of a skill-based intervention for victims of bullying in Brazil. Int. J. Environ. Res. Public Health.

[56] Liu M.J., Ma L.Y., Chou W.J., Chen Y.M., Liu T.L., Hsiao R.C., Hu H.F., Yen C.F. (2018). Effects of theory of mind performance training on reducing bullying involvement in children and adolescents with high-functioning autism spectrum disorder. PLoS ONE.

[57] James A.C., James G., Cowdrey F.A., Soler A., Choke A. (2015). Cognitive behavioural therapy for anxiety disorders in children and adolescents. Cochrane Database Syst. Rev..

[58] Banneyer K.N., Bonin L., Price K., Goodman W.K., Storch E.A. (2018). Cognitive behavioral therapy for childhood anxiety disorders: A review of recent advances. Curr. Psychiatry Rep..

[59] Sciberras E., Mulraney M., Anderson V., Rapee R.M., Nicholson J.M., Efron D., Lee K., Markopoulos Z., Hiscock H. (2018). Managing anxiety in children with ADHD using cognitive-behavioural therapy: A pilot randomised controlled trial. J. Atten. Disord..

[60] Skeen S., Laurenzi C.A., Gordon S.L., du Toit S., Tomlinson M., Dua T., Fleischmann A., Kohl K., Ross D., Servili C. (2019). Adolescent mental health program components and behavior risk reduction: A meta-analysis. Pediatrics.

[61] Navarro R., Serna C., Martínez V., Ruiz-Oliva R. (2015). The role of Internet use and parental mediation on cyberbullying victimization among Spanish children from rural public schools. Eur. J. Investig. Health Psychol. Educ..

[62] Hutson E., Kelly S., Militello L.K. (2018). Systematic review of cyberbullying interventions for youth and parents with implications for evidence-based practice. Worldviews Evid. Based Nurs..

[63] Novin S., Broekhof E., Rieffe C. (2019). Bidirectional relationships between bullying, victimization and emotion experience in boys with and without autism. Autism.

[64] Becker S.P., Mehari K.R., Langberg J.M., Evans S.W. (2017). Rates of peer victimization in young adolescents with ADHD and associations with internalizing symptoms and self-esteem. Eur. Child Adolesc. Psychiatry.

[65] Fekkes M., Pijpers F.I.M., Verloove-Vanhorick S.P. (2004). Bullying behavior and associations with psychosomatic complaints and depression in victims. J. Pediatr..

[66] Kaltiala-Heino R., Rimpelä M., Marttunen M., Rimpelä A., Ratenen P. (1999). Bullying, depression and suicidal ideation in Finnish adolescents: School survey. BMJ.

[67] Nansel T.R., Craig W., Overpeck M.D., Saluja G., Ruan W.J. (2004). Health Behaviour in School-aged Children Bullying Analyses Working Group. Cross-national consistency in the relationship between bullying behaviors and psychosocial adjustment. Arch. Pediatr. Adolesc. Med..

[68] Yen C.F., Yang P., Wang P.W., Lin H.C., Liu T.L., Wu Y.Y., Tang T.C. (2014). Association between school bullying levels/types and mental health problems among Taiwanese adolescents. Compr. Psychiatry.

